# *Dasyrhynchus giganteus* plerocercoids encysting in the musculature of Indian halibut (*Psettodes erumei*): seasonal prevalence, morpho-molecular characterization, and histopathological alterations

**DOI:** 10.1186/s12917-024-04156-y

**Published:** 2024-07-22

**Authors:** Mustafa M. Ibrahim, Hanadi B. Baghdadi, Khalid Shahin, Mostafa Abdel-Glil, Hasnaa Thabit, Marwa M. Attia, Mohamed Abdelsalam

**Affiliations:** 1 Department of Pathology, Animal Health Research Institute, Dokki, Giza, 12618 Egypt; 2 Department of Biology, Faculty of Science, Imam Abdul Rahman Bin Faisal University, Dammam, Saudi Arabia; 3 National Institute of Oceanography and Fisheries, NIOF, 101 Kasr El Ainy St, Cairo, Egypt; 4 Department of Pathology, Faculty of Veterinary Medicine, Zagazig University, Zagazig, Sharkia 44511 Egypt; 5 Department of Zoology and Entomology, Faculty of Science, Assiut University, Assiut, 71526 Egypt; 6 Department of Parasitology, Faculty of Veterinary Medicine, Cairo University, Giza, 12211 Egypt; 7 Department of Aquatic Animal Medicine and Management, Faculty of Veterinary Medicine, Cairo University, Giza, 12211 Egypt

**Keywords:** *Dasyrhynchus giganteus*, Plerocercoids, *Psettodes erumei*, Indian halibut, Prevalence, Seasonal variation, Molecular identification, 28S rDNA, Phylogenetics, Histopathology

## Abstract

This study investigated the prevalence, morphology, molecular identification, and histopathological effects of larval tapeworms (plerocercoids) infecting the skeletal muscles of the Indian halibut (*Psettodes erumei*) collected from the coastal waters of the Arabian Gulf. Numerous oval or round blastocysts, measuring 13–26 mm, were found embedded within the muscular tissues of the Indian halibut, rendering the fish unsuitable for human consumption. Morphological and molecular analyses identified the plerocercoids as *Dasyrhynchus giganteus* (family Dasyrhynchidae), with an overall prevalence of 15.4%. The seasonal prevalence was the highest in summer (14.6%), followed by spring (10.6%), winter (4.4%), and autumn (3.5%). Infection rates increased with fish size. Histopathological examination revealed fibrous connective tissue capsules surrounding the larvae, causing muscular atrophy and degenerative changes, with few inflammatory eosinophilic cells. Molecular and phylogenetic analysis of the 28S rDNA gene sequences confirmed the specimens as *D. giganteus*, clustered closely with other sequences of *D. giganteus* with 100% bootstrap values. This study provided valuable insights into the parasitic infection dynamics, seasonal variation, molecular identification, and histopathological effects, highlighting the importance of monitoring fish for food safety and public health implications.

## Introduction

The Arabian Gulf, a shallow and semi-enclosed water body situated in a dry climatic zone, plays a pivotal role in sustaining marine life, coastal communities, and contributing to global ecological and economic well-being. However, its biological importance is threatened by pollution from industrial activities, which endangers its marine biodiversity [7]. The Jubail province, located along the Saudi Arabian Gulf, is a major fishing center recognized for its abundance of commercially valuable flatfish species, notably the Indian halibut, *Psettodes erumei* [28]. This piscivorous member of the Psettodidae family is widely distributed in Jubail fish markets and throughout other Arabian Gulf nations, owing to its significant commercial value [29].

Fish parasites, particularly Trypanorhynch cestodes, constitute a significant component of marine biodiversity and have the potential to negatively impact fish populations [12]. The order Trypanorhyncha comprises a group of marine tapeworms with over 270 species exhibiting a tripartite life cycle involving a final host (elasmobranch fish), a first intermediate host (small crustacean), and a second intermediate host (teleost fish or invertebrate) [13, 16–19]. The plerocercoid (larval stages) of trypanorhynchus exhibit a lower degree of host specificity and encyst in the visceral organs and muscles of marine teleosts, which can serve as intermediate or paratenic hosts until they are consumed by elasmobranch fish, facilitating exocystation and subsequent development into mature trypanorhyncha [30, 49, 56, 57].

The family Dasyrhynchidae (Cestoda: Trypanorhyncha) contains only one genus, *Dasyrhynchus*, characterized by a large scolex with two bothria and four tentacles in each larva, poeciloacanthous tentacular armature with double chainette, and two or four rows of intercalary hooks on one side of the chainette [14, 47]. The bothria apparatus aids mobility, while the tentacles serve as holdfasts, attaching to the infected host tissues [38, 48]. *Dasyrhynchus giganteus* and *D. variouncinatus*, two species within the genus *Dasyrhynchus*, exhibit similar larval morphological appearances, but the mature form of *D. giganteus* can be distinguished by the presence of two rows of hooks instead of four [15, 27]. *D. giganteus* is found in the tropical part of the Atlantic Ocean and is known to infest fish flesh with its large blastocysts [26].

Infestation of fish by Trypanorhyncha can result in severe damage due to plerocercoid migration, leading to decreased fertility and longevity of the fish [21–23]. Moreover, the presence of blastocysts in fish muscles or the abdominal cavity reduces marketability and causes significant economic losses [46]. Raw fish harboring larval cestode can also cause allergic reactions when accidentally ingested by human consumers [48]. Therefore, it is crucial to perform both parasitological and histopathological investigations on fish sourced from natural fisheries to ensure their safety. Identifying plerocercoids through traditional morphological criteria can be challenging due to the difficulty in distinguishing their adult features. The utilization of molecular tools for the identification and characterization of fish parasites holds significant importance in enhancing accuracy and precision in taxonomic classification and understanding parasite-host interactions. Several reports have highlighted the diagnostic importance of 28S rDNA in fish parasitology, serving as a reliable genetic marker for elucidating the phylogenetic relationships and taxonomic classification of Trypanorhyncha, aiding in accurate species identification and understanding their evolutionary history [41].

Despite several studies conducted on trypanorhynchids from marine fish inhabiting the Red Sea and Arabian Gulf [2–5, 8, 10], there is a lack of information on the presence of *D. giganteus* plerocercoids in *P. erumei* along the coastlines of the Arabian Gulf of Saudi Arabia [6, 9, 32]. The present study aimed to investigate the occurrence and seasonal prevalence of Trypanorhyncha species infection in the skeletal musculature of Indian halibut obtained from the Eastern coastal region of the Saudi Arabian Gulf over a period of one year. Meanwhile, morphological and genomic characteristics of the recovered parasite and the associated histopathology were elucidated.

## Materials and methods

### Ethical approval

All experimental fish protocols followed the guidelines and the Institutional Animal and Care and Use Committee of Assiut University (No. Vet AU 04–2024-300383).

### Fish sampling

A total of 350 fresh large-sized Indian halibut (known locally as El-Khaufa), comprising 150 in summer, 75 in spring, 45 in winter, and 80 in autumn, were collected over a period of one year from January to December 2019 as part of the routine clinical inspection at Jubail fish markets for this study. Fish specimens were captured using the bottom trawl method and then their total length and weight were measured. Fish were transferred in an insulated ice box with minimal delay to the laboratory at the fish welfare unit in Jubail province, where gross and post-mortem examinations were conducted. Upon arrival, a thorough clinical examination was conducted to check for any signs of diseases.

### Parasitological examination

Fish were dissected and examined for parasitic infection. A stereoscopic dissecting microscope was used to examine the body cavity, internal organs, and skeletal muscles of the sampled fish. The encapsulated plerocercoids were removed from the infected muscle layer, and the juvenile scolices in the larval stage were isolated by opening the walls of blastocysts [62]. The recovered larvae were cleaned, rinsed, and stored in 70% ethanol. Larvae were submerged in distilled water, allowing for the tentacles to move out of the Bulbs towards the head region through contractile movement. The larval stages were firmly placed between two glass slides and examined under a light microscope. The larval stages were then fixed in 10% formalin and stained with carmine stain [54], and the identification process was carried out following the method of Palm [48] and Palm et al. [50].

### Molecular identification

#### DNA extraction

DNA was extracted from plerocercoids retrieved from infected fish; collected from the same locality in different seasons of the year; using the Gene JET Genomic DNA purification kit (Thermo Fisher Scientific, UK) following manufacturer instructions. The genomic DNA was quantified using the Implen Nano Photometer (NP80, Munich, Germany), followed by normalization to a concentration of 50 ng/μL that was stored at − 20 °C till processed.

#### PCR amplification of 28S rDNA

PCR amplification of the D1-D2 region of the 28S rDNA was performed using the forward primer LSU-5: 5'-TAGGTCGACCCGCTGAAYTTAAGCA-3' and the reverse primer 1500R: 5'-GCTATCCTGAGGGAAACTTCG-3' [41, 42]. PCR was conducted in a 25-μL reaction volume containing 12.5 μL of 2X MyTaq HS Red Mix (Bioline, MA, USA), 2μL of genomic DNA extract, 1 μL of each primer (10 ng) and DEPC water to the volume. The PCR cycling conditions included: an initial phase of denaturation at 95 °C for 5 min, 40 cycles at 95 °C for 30 s, annealing at 55 °C for 30 s, a final extension at 72 °C for 10 min followed by 1 cycle at 4 °C for 10 min. The PCR products were visualized following electrophoresis using 1% agarose gel. Purification of the PCR amplicons was performed using the QIAquick Gel Extraction Kit (QIAGEN).

#### Sequencing and phylogenetic analyses

Sequencing of the purified amplicons was bidirectionally conducted using the Big Dye® Terminator v3. 1 Cycle Sequencing Kit (Applied Biosystems, USA) along with the same primers used in the PCR process. The sequences were subsequently subjected to capillary electrophoresis utilizing the ABI PRISM3130 automated sequencers (Applied Biosystems, USA). The raw sequences were manually edited using a sequence alignment editor; BioEdit v. 7.2.5 [31]. The assembled contigs were subsequently aligned with respect to the corresponding *28S rDNA* sequences present in the GenBank database, using the BLASTN tool service. The produced nucleotide sequences were submitted to the GenBank nucleotide database for assigning specific accession numbers. A phylogenetic analysis was performed for comparison of the partial sequences of *28S rDNA* from this study to 29 genes sequences representing various families of Trypanorhyncha cestodes, including Family: Dasyrhynchidae (*D. giganteus* and *D. variouncinatus*; Family: Lacistorhynchidae (*Callitetrarhynchus gracilis; Paragrillotia similis; Pseudogilquinia pillersi; Pseudolacistorhynchus heroniensis; Lacistorhynchus dollfusi; Grillotia rowei; Floriceps minacanthus*; and *F. saccatus*); Family: Mustelicolidae (*Diesingium lomentaceum*); Family: Otobothriidae (*Otobothriumprope cysticum; Symbothriorhynchus tigaminacantha; Proemotobothrium linstowi*; *Fossobothrium perplexum*; and *Ancipirhynchusa fossalis*); Family: Pterobothriidae (*Pterobothriumplatycephalum*); Family:Gilquiniidae (*Gilquiniarobertsoni* and *Vittirhynchus squali*); Family: Gymnorhynchidae (Gymnorhynchusisuri; and *Molicolathyristes*); Family: Hornelliellidae (*Hornelliella annandalei*); Family:Sphyriocephalidae (*Hepatoxylon trichiurid*; *Sphyriocephalus viridis; Heterosphyrio cephalustergestinus; H. oheolumiae*); Family: Aporhynchidae (*Aporhynchus norvegicus*); and Family: Tetraphyllidea (*Acanthobothrium zimmeri*). Firstly, a multiple sequence alignment was performed using the Clustal W program. The maximum likelihood (ML) and Bayesian inference (BI) methods were used to establish the phylogenetic relationship. The maximum likelihood analysis was employed using MEGA11 [59]. The ML parameters were determined by employing the general time reversible model which incorporates a gamma-distributed rate in conjunction with invariant sites (GTR + G + I). The selection of this model was based upon its possession of the lowest score as determined by the Bayesian information criterion (BIC) and corrected Akaike information criterion (AIC), as well as high bootstrap confidence values, which were obtained through 1,000 replicates. On the other hand, Bayesian analyses were conducted using Mr Bayes v3.2.6 [35].

#### Histopathology

Specimens of the infected muscles harboring parasitic cysts were excised and fixed overnight in 10% neutral buffered formalin (Sigma-Aldrich, Cambridge, UK). The fixed samples were embedded in paraffin. The blocks of paraffin were cut into sections with a thickness of 5 μm, subsequently stained with hematoxylin and eosin (H & E, Abcam, UK), and finally analyzed using a light microscope (Olympus BX50, Japan) [11, 36].

## Results

### Clinical examination

**Fig. 1:**
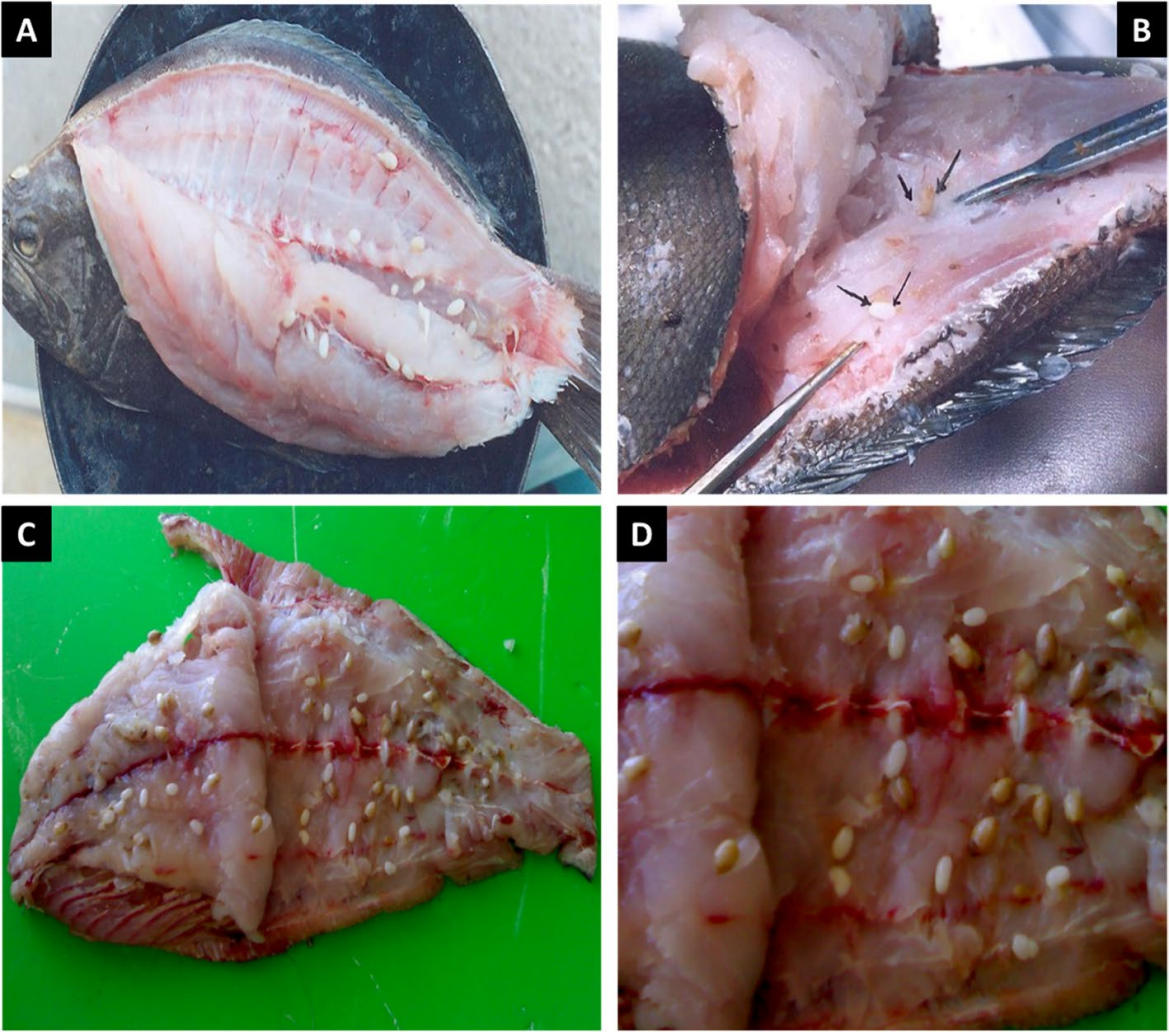
Indian halibut infected with plerocercoid larvae of *D. giganteus*. **A**, **B** Mild infected, (**C**, **D**) heavy infected fish showing plerocercoid larvae attached to muscle following sagittal incision

The collected Indian halibut samples ranged in size from 20 to 60 cm, with an average weight of 500 g to 3.5 kg. During the gross examination, the fish specimens did not exhibit any noticeable external abnormalities. However, during the post-mortem examination, numerous parasitic blastocysts were found to be extensively embedded within the muscular tissues, rendering the fish unacceptable for human consumption. The observed blastocysts were identified by their distinct oval or round shape, measuring 13–26 (17 ± 1.5) mm in length, and varying in color from milky white to yellowish or dark brown. The blastocysts were composed of larva-filled structures, accompanied by minute quantities of serous fluid **(**Fig. 1).

### Prevalence and seasonal variation

**Fig. 2:**
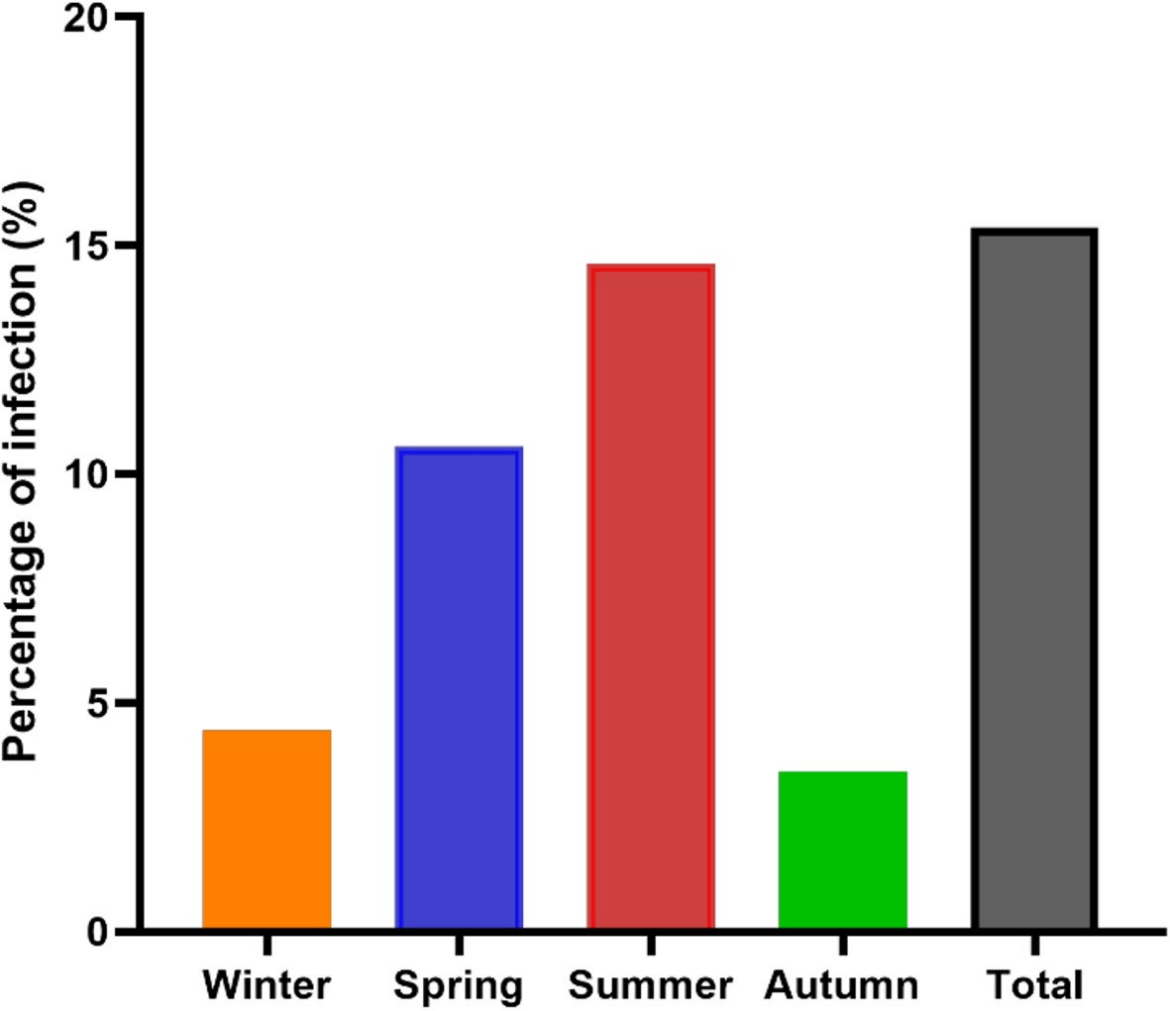
Seasonal variation of *D. giganteus* in infected Indian halibut from Saudi Arabian Gulf

Trypanorhyncha blastocysts were recognized in 45 out of 350 fish samples, representing an overall prevalence of 15.4%. The prevalence of infection fluctuated according to seasonal variation (Fig. 2). The seasonal prevalence of Trypanorhyncha spp. infection was the highest in summer at 14.6% (22/150), followed by 10.6% in spring (8/75), 4.4% in winter (2/45), and 3.5% in autumn (3/80). The prevalence of infection rate in the muscles of infected fish varied depending on the size of the infected fish, with higher infection rates associated with increased fish size (Table 1).

**Table 1:** Intensity of infection of blastocysts of *D. giganteus* infection based on the size of the infected Indian halibut

Size of the infected fish	No. of blastocysts
Less than 20 cm	Not detected
20 cm to 30 cm	30—40 cysts
30 cm to 40 cm	100 – 150 cysts
40 cm to 60 cm	200 – 250 cysts

### Morphological description of *D. giganteus*

Based on the examination of 10 specimens, the plerocercoid stage exhibited prominent oval blastocysts, measuring 6.9–15.5 (10.9 ± 0.59) mm in length, and contained a scolex. The overall length of the scolex was between 5.3–6.6 (5.5 ± 0.50) mm. The length of the two bothria on the post-larva were measured to be 145–175 (165 ± 2.5) mm, exhibiting prominently thick edges. The posterior border of each bothridium exhibited two prominent fossettes, characterized by their enlarged size, possessing a short tail and a striated body.

Each plerocercoid possessed four elongated tentacles that terminated in a pointed end, supported by several rows of hooks and encased in spiral-shaped sheaths measuring 1.50–1.95 (1.55 mm ± 0.5) in length. The lengths of the bulbs ranged between 1.55 and 1.89 (1.59 ± 0.5) mm. The identified blastocysts showed varying degrees of pigmentation, ranging from milky white to a gradient of yellowish to dark brown.

### Phylogenetic analyses

The 28S rDNA gene sequences of the plerocercoids were obtained from four fish specimens collected from the same geographical area but during different seasons throughout the year. These sequences yielded identical DNA fragments with a length of 1391 base pairs. The 28S rDNA gene sequences were assigned to GenBank accession numbers OR105010, OR378532, OR378533, and OR378534. Based on a thorough analysis of the DNA sequence fragments and their alignment to existing records in GenBank, it was confirmed that these recovered plerocercoid specimens belong to the taxonomic genus *Dasyrhynchus* and have been conclusively identified as *D. giganteus*.

**Fig. 3:**
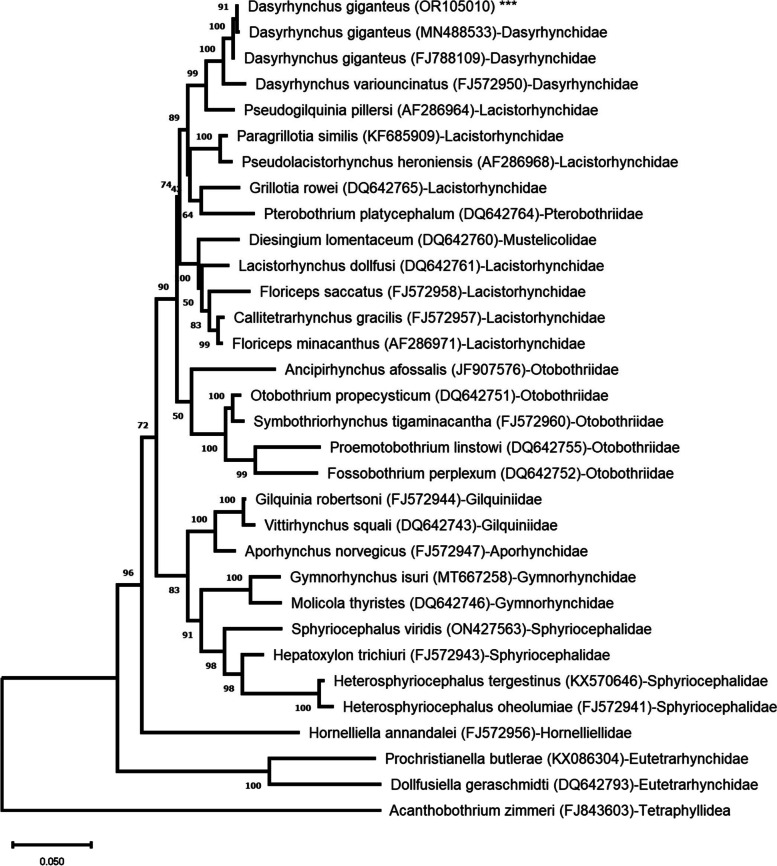
A phylogenetic tree derived from *28S rDNA gene* analysis through Bayesian inference illustrated the relationship between the current *D. giganteus* and other Trypanorhyncha species. The evolutionary history was inferred by using the Maximum Likelihood method and General Time Reversible model. The percentage of trees in which the associated taxa clustered together is shown next to the branches. 28S rDNA sequence from this study is highlighted by asterisks

The 28S rDNA sequences from this study showed 99.84—99.61% similarity to *D. giganteus* (GenBank accession numbers MN488533.1 and FJ788109.1). The phylogenetic tree, derived from the post-burn in trees of the two Bayesian runs accomplished by MrBayes, illustrated that the plerocerci of *D. giganteus* located in *P. erumei* were clustered within a group of other *D. giganteus* with 100% high bootstrap values (Fig. 3). This branch, representing *D. giganteus*, was grouped with *D. variouncinatus* and formed a strongly supported monophyletic group, as demonstrated by very high bootstrap values of 99%.

### Taxonomic summary

The parasites identified in this study belong to the species *D. giganteus* (Family: Dasyrhynchidae). They were found infecting the Indian halibut, *P. erumei* (Family: Psettodidae), collected from the coasts of the Saudi Arabian Gulf site in the Jubail province, Saudi Arabia. The plerocercoid larvae were embedded within the skeletal muscles of the infected fish.

**Parasites name**: *D. giganteus.*

**Family**: Dasyrhynchidae.

**Host:** Indian halibut *P. erumei* (Family: Psettodidae).

**Locality**: Coasts of Saudi Arabian Gulf site, Jubail province, Saudi Arabia.

**Site of infection:** plerocercoid larvae were found embedded within the skeletal muscles of infected fish.

### Histopathological examination

**Fig. 4:**
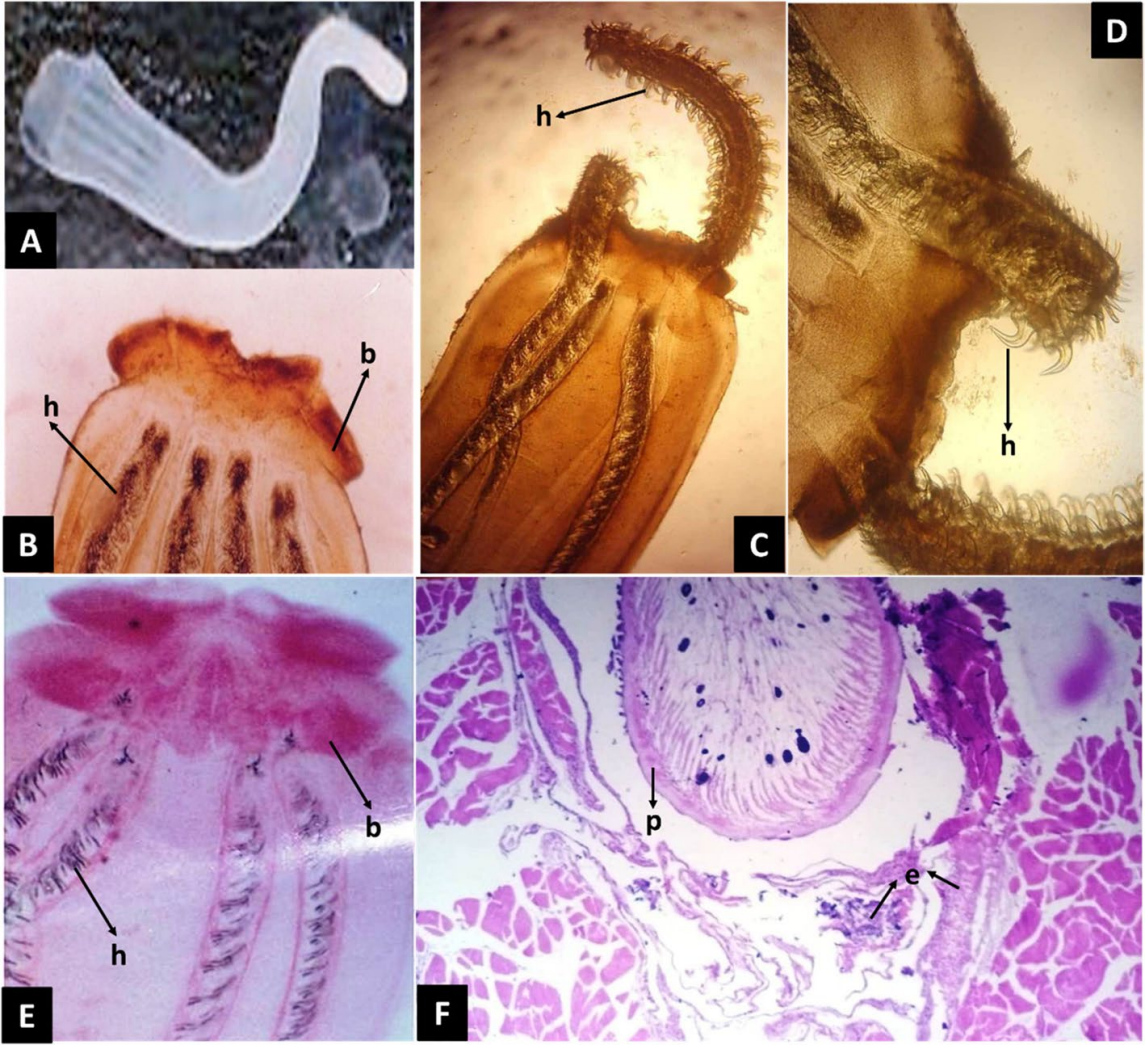
Morphology of *D. giganteus* and associated pathology in infected Indian halibut from Gulf of Saudi Arabia. **A*** D. giganteus* plerocercoid extracted from the blastocyst. **B** anterior end of the larval stage showing the bothridia (b): bulbous; (h): hooks). **C** and **D** protruded proboscis with hooks (h). **E** Anterior end stained with carmine showed bulbous (b) and hooks (h). **F** Histopathological section of infected muscles showing the capsular layer of the parasites (p) associated with pressure atrophy in muscles and degenerative changes in the adjacent surrounding muscles and scanty inflammatory cells of eosinophilic cells (e)

The morphology of the capsule was characterized by a distinct bladder-like or elongated appearance, typically displaying a white coloration. However, older capsule blastocysts demonstrated a color shift from white to yellowish or blue-black, accompanied by a slight iridescence. Histological examination revealed that infected fish had enclosed blastocysts filled with larvae in their muscular tissues. These cysts showed fibrous connective tissue in the capsule layer, leading to muscular atrophy and degenerative changes in the adjacent surrounding muscles. Additionally, only a few inflammatory cells, mainly eosinophilic cells, were reported (Fig. 4).

## Discussion

Trypanorhynch cestode infections in fish are a significant concern due to their multifaceted impacts on fish health, human health, and economic implications. These parasites not only affect the vitality of fish populations but also pose potential risks of zoonotic transmission to humans. The economic consequences of trypanorhynch cestode infections in farmed and wild fish can be substantial, leading to reduced growth rates, increased mortality, and lower-quality fish products. The present study reports on the seasonal prevalence of infection by a trypanorhyncha metacestode in the Indian halibut (*P. erumei*), which is considered one of the major demersal fish species in Arabian Gulf fisheries. Trypanorhynch cestodes of various species have been previously reported in the Arabian Gulf and Oman Sea, infecting a range of aquatic organisms, including elasmobranch species [34], greasy grouper (*Epinephelus tauvina*), yellowfin hind (*Cephalopholis hemistiktos*), green snapper [32], and even shrimp (*Penaeus semisulcatus*) [40], causing significant muscular pathology represented mainly by inflammatory responses and atrophy [32].

*D. giganteus*, a member of the trypanorhynch cestode family Dasyrhynchidae, is known to infect various teleost species, including common jack (*Caranx hippos*) and leatherjacket fish (*Oligoplites saurus*) in Brazil, greater amberjack (*Seriola dumerili*) in the USA, and swordfish (*Xiphias gladius*) in the northwest Atlantic [61]. The detection of *D. giganteus* in different teleost species from diverse geographic locations in the Atlantic Ocean and Arabian Gulf implies a broad geographic spread of this parasite and a degree of non-specificity regarding its fish hosts. In the current study, the recovered plerocercoid from Indian halibut has been identified as *D. giganteus* based on morphological and molecular analyses. This difference in prevalence may be attributed to variations in the abundance of the first intermediate host between the distinct geographical locations of the studies. Although the infected Indian halibut examined in this study displayed no evident aberrant clinical signs, post-mortem analysis of the muscular layer revealed the presence of trypanorhyncha plerocercoids with varying shapes, sizes, and colors within the muscle tissue. The yellowish encapsulations found in the fish flesh infected by trypanorhyncha plerocercoids suggest that the parasitic infection has existed for a minimum of two years [25, 45]. Additionally, our investigation indicates a seasonal variation in the infection rate, with the highest infection rates observed during the summer and spring months, which aligns with previous records from Hassan et al. [32]. This seasonal fluctuation can be attributed to the availability of elasmobranch definitive hosts and natural changes in environmental conditions that affect the abundance of suitable crustacea (first intermediate hosts) consumed by the Indian halibut.

The analysis of 28S rDNA gene sequencing employed in this study has proven to be a valuable technique for the identification and differentiation of *D. giganteus*, supporting the findings of Olson et al. [43], who utilized 28S rDNA sequencing to identify trypanorhyncha cestoda retrieved from infected fish. The current *D. giganteus* isolates exhibited the highest percentage of similarity when compared to other *D. giganteus* sequences, further verifying its identity based on both morphological and molecular analyses. Several studies have highlighted the utility of 28S rDNA as a reliable genetic marker for elucidating the phylogenetic relationships and taxonomic classification of trypanorhynchs and other fish parasites [33, 44, 63]. The use of molecular tools, such as DNA barcoding and sequence analysis, has become increasingly important in addressing the challenges associated with traditional morphological identification, particularly for larval stages or cryptic species [20, 58]. The integration of molecular diagnostics with classical morphological techniques has proven to be a powerful approach in fish parasitology, providing insights into host-parasite interactions, understanding the life cycles and transmission dynamics of parasites, and facilitating accurate species identification [53, 60]. Furthermore, molecular methods have contributed to the discovery of new parasite species and the re-evaluation of existing taxonomic classifications [1, 24, 37].

Histopathological examination revealed that *D. giganteus* plerocercoids initiate a progressive and chronic response characterized by an extensive and pronounced fibrotic reaction, leading to the encapsulation of the plerocercoid cysts within the muscular tissues. This encapsulation process typically induces atrophy within the surrounding muscular tissues, as previously reported by Hassan et al. [32]. Our findings corroborate these observations, with the presence of parasitic capsules and pressure atrophy in muscles, as well as degenerative and cellular changes in adjacent tissues. These observations may be attributed to an immune response mechanism from the infected fish, initiating the formation of fibrous capsules and eosinophilic cell infiltration to control the invading parasites.

Fish parasitic infections are significant for human health due to the potential transmission of pathogens from infected fish to humans, leading to various health concerns including gastrointestinal disorders and allergic reactions. Our findings indicated that the flesh of the Indian halibut was rejected due to significant infection with the plerocercoid of *D. giganteus*. Several previous studies have revealed that the presence of Trypanorhyncha plerocercoids in fish flesh can have deleterious effects, rendering it unsuitable for consumption [55, 57]. Furthermore, the presence of Trypanorhyncha plerocercoids in the flesh can lead to the deterioration and softening of muscular tissues, caused by the proteolytic activity exerted by proteins during larval invasion [51]. Trypanorhyncha plerocercoids present potential hazardous effects and constitute a significant zoonotic concern to humans, particularly through accidental ingestion. Although there is no conclusive evidence of their harmful impact on human health [39], certain species of Trypanorhyncha plerocercoid have been found to cause allergic reactions in humans [52].

## Conclusion

To the best of our knowledge, the current study represents the first record of *D. giganteus* plerocercoids invading the muscle tissues of Indian halibut in the Jubail province along the Eastern Arabian Gulf coastlines. By employing morphological, histological, and molecular approaches, we successfully deciphered the characteristic features of the parasite, illustrated the associated tissue pathology, and confirmed its identity and phylogenetic relatedness to closely related parasites. The detection of high numbers of *D. giganteus* plerocercoids within the muscle tissues of Indian halibut can render the fish unsuitable for consumption. To ensure the safety of consuming fish, a thorough inspection of the fillet for the presence of any parasites and detailed characterization and identification are warranted. Proper freezing and cooking techniques should also be employed to reduce potential health risks. Our study highlights the need for maintaining hygienic conditions in commercial fish markets and improving education for those involved in the inspection of seafood origin.

## Data Availability

The 28S rDNA gene sequences of *Dasyrhynchus giganteus* plerocercoids derived from this study were submitted to the GenBank database and assigned accession numbers: OR105010, OR378532, OR378533, and OR378534.
